# Quality filtering of Illumina index reads mitigates sample cross-talk

**DOI:** 10.1186/s12864-016-3217-x

**Published:** 2016-11-04

**Authors:** Erik Scott Wright, Kalin Horen Vetsigian

**Affiliations:** 1 Department of Bacteriology, UW-Madison, Madison, USA; 2 Wisconsin Institute for Discovery, UW-Madison, 330 N. Orchard St, Madison, 53715 WI USA

**Keywords:** Illumina, Sequencing, Multiplexing, Sequencing errors

## Abstract

**Background:**

Multiplexing multiple samples during Illumina sequencing is a common practice and is rapidly growing in importance as the throughput of the platform increases. Misassignments during de-multiplexing, where sequences are associated with the wrong sample, are an overlooked error mode on the Illumina sequencing platform. This results in a low rate of cross-talk among multiplexed samples and can cause detrimental effects in studies requiring the detection of rare variants or when multiplexing a large number of samples.

**Results:**

We observed rates of cross-talk averaging 0.24 % when multiplexing 14 different samples with unique i5 and i7 index sequences. This cross-talk rate corresponded to 254,632 misassigned reads on a single lane of the Illumina HiSeq 2500. Notably, all types of misassignment occur at similar rates: incorrect i5, incorrect i7, and incorrect sequence reads. We demonstrate that misassignments can be nearly eliminated by quality filtering of index reads while preserving about 90 % of the original sequences.

**Conclusions:**

Cross-talk among multiplexed samples is a significant error mode on the Illumina platform, especially if samples are only separated by a single unique index. Quality filtering of index sequences offers an effective solution to minimizing cross-talk among samples. Furthermore, we propose a straightforward method for verifying the extent of cross-talk between samples and optimizing quality score thresholds that does not require additional control samples and can even be performed *post hoc* on previous runs.

**Electronic supplementary material:**

The online version of this article (doi:10.1186/s12864-016-3217-x) contains supplementary material, which is available to authorized users.

## Background

In recent years Illumina sequencing has emerged as a mainstay for numerous biological applications. Due to the immense number of sequences that can be obtained, it is often useful to sequence DNA from multiple samples in a single run. This multiplexing process relies upon unique “index” sequences, termed i5 and i7, that are added to both sides of the DNA being sequenced. With only a few unique i5 and i7 sequences, hundreds of different i5 and i7 combinations can be created, enabling many samples to be simultaneously sequenced. De-multiplexing the samples after sequencing only requires finding the sequencing reads associated with each index pair that was added to the sequencing run.

As with other sequencing approaches, the Illumina method has been characterized for the frequency and type of errors that are generated [1]. Substitutions, where one base is misread as another, are the most frequent error class and occur more often toward the end of the sequence [2, 3]. Insertions, deletions and motif-specific errors occur less frequently, but they can still cause problems for certain applications [4, 5].

Another type of error involves cross-talk among multiplexed samples and has received far less attention despite recent reports that error rates can be significant [6–8]. Such errors are particularly insidious in applications that require the detection of variants that are rare in one sample but abundant in others, which includes biosphere surveys, investigations of ancient DNA, and the identification of cancerous cells [8]. Cross-talk errors can also be problematic if a large number of samples are multiplexed, such that each sample is a small fraction of the total number of reads. Since cross-talk can come from multiple sources, it has sometimes been attributed to experimental mistakes, cross-contamination during primer synthesis, multiple misread bases within index sequences, or sample carryover from previous sequencing runs on the same machine [9].

**Fig. 1:**
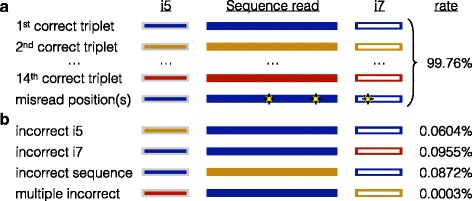
Rates of different misassignment errors on the Illumina platform. **a** Unique index and read sequences that were well separated in sequence space (colored rectangles) were used to form 14 distinct samples and multiplexed in the same Illumina sequencing run. Misread bases (yellow stars) make up the most common error type, but are still attributable to their correct triplet. **b** Misassigned reads appear as unexpected triplets, and can be categorized as either index misassignments (0.16 % total) or sequence misassignments (0.09 %)

In view of the increasing importance of multiplexing on the Illumina platform, we systematically investigated cross-talk errors in order to rule out certain causes and determine whether there are any satisfactory solutions to the problem. To this end, we constructed 14 unique combinations of i5 indices, i7 indices, and read sequences (Fig. 1a) while carefully controlling for potential sources of cross-contamination such as primer synthesis. The sequences of all reads were well-separated in sequence space to minimize cross-talk due to misread bases. Surprisingly, we observed that cross-talk was due to three different types of misassignments (Fig. 1b) that occurred at similar rates. Furthermore, we found that quality filtering of the index pairs was sufficient to all but eliminate misassignments between samples without sacrificing a substantial fraction of reads.

## Results

Using standard de-multiplexing protocols, we observed a 0.09 % rate of *sequence misassignments*, which have the correct i5 and i7 index pair but incorrect sequence, and a 0.16 % rate of *index misassignments*, which have a correct sequence read but a single incorrect i5 or i7 index. These rates are consistent with prior studies that found misassignment rates between 0.06 and 0.21 % [8, 9]. Furthermore, the rate of sequence misassignment was similar to that of i5 or i7 index misassignment (Fig. 1b), indicating that the sequence is being misassigned rather than both index sequences being independently misassigned. Both sequence and index misassignments will contribute to cross-talk between samples when each sample is separated from other samples by a single index, whereas only sequence misassignments are relevant when unique dual-indexing is used. Nevertheless, the existence of sequence misassignments indicates that even the use of two unique index sequences is insufficient to eliminate cross-talk.

**Fig. 2:**
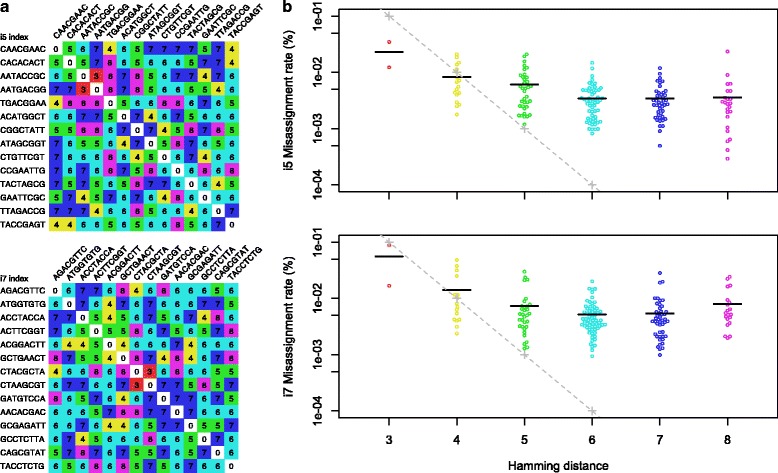
Misassignment rates were weakly correlated with the hamming distance between index sequences. **a** Matrices showing the hamming distance between i5 and i7 index sequences used in this study. **b** The rate of triplets with an incorrect i5 (or i7) index as a function of the hamming distance to the correct i5 (or i7) index. Horizontal lines indicate the mean misassignment rate at each hamming distance. Note the log-scale y-axis. The theoretical misassignment rates based on independent substitutions are shown in gray for an exaggerated 10 % substitution rate (Q10); lower substitution rates would simply shift the dashed-line to the left. The observed misassignment rate does not decrease exponentially as would be expected if misread errors are independent, indicating that misread bases are not the cause of misassignment errors

Misassignments can in principle result from multiple misread bases within an index sequence. However, even at a high average error rate of 1 % (Q20), the chance of at least three positions being misread is 10^−6^ assuming that errors are independent. The observed rate of index misassignment was far greater than expected, regardless of the number of differences between index sequences (Fig. 2). If two unique index sequences are used, the probability of both the i5 and i7 being misread as another index pair is expected to be around 10^−12^. Therefore, since we obtained approximately 10 million reads per sample, we would expect zero sequence misassignment due to misread bases. To further verify these assumptions, we de-multiplexed another index pair where neither the i5 or i7 index was included in the experiment. There were no reads attributed to this index pair, confirming that the per-base error rate of Illumina sequencing does not explain the observed rate of cross-talk.

**Fig. 3:**
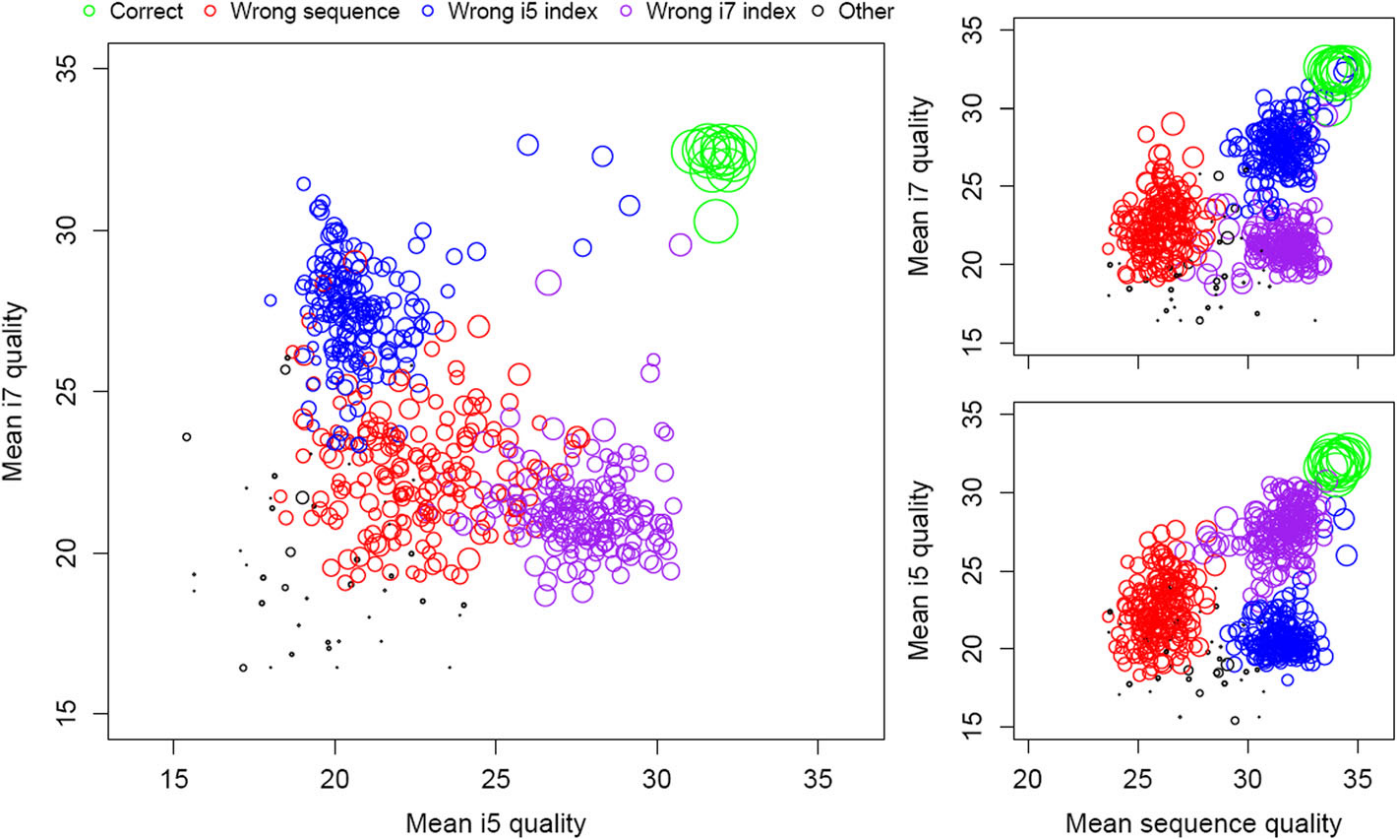
Breakdown of average quality scores by error type. Each point represents the reads obtain for one triplet (i5, i7, and sequence read), and is scaled to the log of the read count. Correct triplets (green) have high quality across all read steps, whereas sequence misassignments have low quality in all three read steps. In contrast, index misassignments tend to have low quality in the step for which they are misassigned

Having ruled out misread bases as the cause of most misassignments, we next investigated whether incorrect reads were associated with low quality scores. Figure 3 shows that correct triplets (i5, i7, and sequence) tended to have high quality in both index read steps, whereas index misassignments tended to be low quality in the step for which they were misassigned. The average quality scores of i5 and i7 index reads appear to be largely independent, i.e. low quality in one does not imply low quality in the other. This may be due to the fact that the two index sequences are read separately after the cluster is inverted on the flow cell. In contrast, sequence misassignments tended to have poor quality i5 and i7 index reads in addition to a low quality sequence read (Fig. 3). Moreover, quality scores were generally lower across the entire length of misassigned reads, rather than only being low quality in a specific region (Additional file 1: Figure S1).

**Fig. 4:**
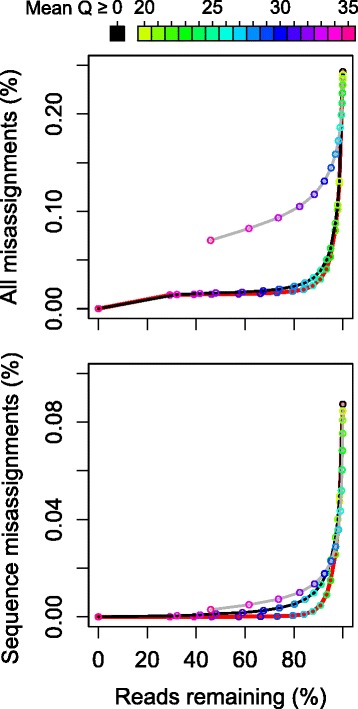
Trade-off between removing misassigned and preserving correct reads during quality filtering. (Top) Misassignments were not efficiently removed by quality filtering the sequence reads (gray line), whereas quality filtering the i5 and i7 index sequences was highly effective (black line). Quality filtering sequence reads in addition to index reads (red line) did not remove substantially more cross-talk. (Bottom) Quality filtering either sequence reads or index reads was effective at removing sequence misassignments

The observed quality score pattern has several implications for filtering incorrect reads. First, filtering low quality sequence reads is expected to be insufficient to eliminate anything other than sequence misassignments. This implies that the i5 and i7 must be quality filtered to eliminate index misassignments. Second, filtering low quality i5 and i7 index reads may be sufficient to eliminate both sequence misassignments and index misassignments without needing to quality filter the sequence read. We tested these hypotheses by applying increasing stringencies of quality score filtering and observing the remaining cross-talk. Here we distinguished between three strategies: quality filtering only the sequences, only the index pairs, and filtering all read steps. As expected, keeping only high quality sequence reads nearly eliminated sequence misassignments but not index misassignments (Fig. 4), whereas filtering the index sequences largely prevented all types of misassignment. By filtering the index reads to an average quality score of ≥ 26 (0.25 % probability of error per base) it was possible to reduce the overall rate of incorrect triplets from 0.24 to 0.03 % while maintaining 88 % of total reads. A combined strategy was only slightly more effective at eliminating both types of misassignment. Thus, quality filtering of index reads provides a simple way to minimize cross-talk while preserving the vast majority of reads.

## Discussion

Misassignment errors could result from distinct cluster originators forming at an overlapping spot on the flow cell [8]. If this were the case, we might expect the quality score profiles of incorrect reads to oscillate between low quality in positions where the two sequence clusters differ (e.g., one A, one C) and high quality where they are identical (e.g., both A). However, we did not observe any such pattern in the quality score signals of incorrect triplets, perhaps because there is a poor correlation between the quality score and the actual probability of error [3] or because neighboring positions are taken into account when assigning quality scores. Nevertheless, we would expect overlapping clusters to lower the quality of all read steps due to competing signals, yet this was also not observed. Instead it appears that one cluster tends to overpower the other during each read step (i5, i7, or sequence), and the overpowering cluster in the pair can switch between read steps.

**Fig. 5:**
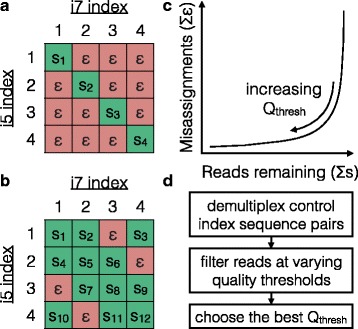
Recommended procedure for removing background reads. **a** When using unique dual index sequences for every sample (s_i_), each missing index pair offers a negative control that provides an estimate of the number of misassigned reads (ε). **b** When almost all index combinations are being used, controls can be added by purposefully omitting samples for some combinations of index sequences. **c** The quality score threshold (Q_thresh_) can then be optimized by plotting the sum of misassignments versus the number of reads remaining. **d** A value of Q_thresh_ can be selected that minimizes misassignments while maximizing the number of reads that remain

While a quality score threshold of 26 was sufficient to eliminate most misassignments in this study, this threshold may vary from run-to-run depending on the run’s overall quality and other factors. For this reason, it may be useful to detect misassignments and then vary the quality score threshold to observe its effect on their removal (Fig. 5). Misassignments can be detected by de-multiplexing index combinations that should not be present in the sequencing run but for which the i5 and i7 index sequences exist separately in other samples. In the absence of misassignments the number of sequences attributable to missing index pairs should be zero. This provides a straightforward method for both verifying misassignments and confirming their removal. Also, this method does not depend upon knowing the sequence variants that belong to each sample.

## Conclusions

To our knowledge, this is the first systematic study of cross-talk on the Illumina platform that uses standard dual indexing as opposed to custom or single indexing schemes. Previous studies of cross-talk identified the advantages of dual indexing over single indexing and of quality filtering index sequences [7, 8]. Here we extended these findings by showing that there are three independent modes of cross-talk: incorrect i5 index, i7 index, and sequence. The existence of sequence misassignments prevents dual indexing from completely eliminating cross-talk without quality filtering. It also means that if only a single (i7) index is used, filtering on sequence quality in addition to index quality is the best strategy. In agreement with previous work [7], we determined that no amount of quality filtering can completely eliminate cross-talk when samples are only separated by one of two index sequences. Thus, unique dual indexing is required when identification of extremely rare variants is critical. We also proposed a simple method for both quantifying cross-talk and choosing run-specific or application-specific thresholds for mitigating it by counting reads assigned to unexpected index pairs during quality filtering (Fig. 5).

Cross-talk between samples effectively limits the number of index pair combinations that can be reliably used. As the fraction of clusters sharing an i5 or i7 increases, the number of misassigned reads will concomitantly increase. Eventually, even at small rates of misassignment the incorrect reads would rise to an intolerable level if enough index combinations were used. This is supported by a previous study in which the rate of cross-talk was estimated to approach 1 % when 625 index pair combinations were used [6]. For this reason, we believe it is necessary to quality filter index reads in addition to the sequencing read when employing a multiplexing strategy. Furthermore, to mitigate the issue of spurious results due to cross-talk in the literature, we recommend that repositories such as the Sequence Read Archive (SRA) [10] enable and encourage the submission of quality scores for index sequences and unexpected (control) index pairs. This would allow retroactive filtering of published sequences, and would also provide a means for automatic accumulation of data on the magnitude of sample cross-talk as sequencing platforms evolve.

## Methods

### Template DNA extraction and PCR amplification

A total of 13 strains (Additional file 1: Table S1) belonging to the genera *Amycolatopsis* or *Streptomyces* were grown at 28 °C in 1 mL of 1/10^th^ concentration ISP2 medium (10 g Malt extract, 4 g Yeast extract, and 4 g Dextrose per 1 L) for 9 days. The remaining protocol closely paralleled that of a previous study [6]. Briefly, the cultures were centrifuged at 1000 rcf for 10 min to pellet the cells. A 700 μL volume of supernatant was removed, the remaining volume was vortexed, and 200 μL of the concentrated mycelium was transferred to a 0.2 mL thin-wall tube (Corning). These tubes were sonicated at 100 % amplitude for 60 s using a Model 505 Sonicator with Cup Horn (QSonica) while the samples were completely enclosed. After sonication, the samples were centrifuged, and the supernatant containing DNA was used as template for PCR amplification.

Extracted DNA was amplified using indexed primers containing adapters (Additional file 1: Table S2). Samples were carefully arranged into a 96 well plate in alternating rows and columns to prevent any possibility of cross-contamination. Primers were designed to target either a stably integrated chromosomal barcode or the RNA polymerase subunit β (*rpoB*) gene. The PCR reaction consisted of a 2 min denaturation step at 95 °C, followed by 40 cycles of 20 s at 98 °C, 15 s at 67 °C, and 15 s at 80 °C. The PCR reaction contained 10 μL of iQ Supermix (Bio-Rad), 0.8 μL of 10 μM forward primer, 0.8 μL of 10 μM reverse primer, 4 μL of DNA template, and 5.9 μL of reagent grade H_2_O per sample. Primers were synthesized by Integrated DNA Technologies using their TruGrade service that is intended to prevent cross-contamination during synthesis. Furthermore, primers were purchased across multiple orders that were staggered in time to further ensure that primer cross-contamination could not occur.

### DNA purification, sequencing, and analysis

PCR products were purified separately with the Wizard SV-Gel and PCR Cleanup System (Promega). Samples were sequenced by the UW-Madison Biotechnology Center on an Illumina Hi-Seq 2500 in rapid mode. Sample concentrations were determined using an Agilent 2100 Bioanalyzer, and pooled immediately prior to sequencing in order to reach a target density of 8.5e5 to 1e6 clusters per mm^2^. Spiking PhiX was unnecessary because the sequences’ first 5 bases were well separated (hamming distance from 2 to 5), and we have not noticed a reduction in cross-talk from adding PhiX in prior runs. Single-end sequencing was performed for 51 cycles. After sequencing the cluster density was determined to be 9.9e5/mm^2^.

Samples were de-multiplexed using Illumina’s bcl2fastq (v2.17) software and its associated defaults, that is, allowing 1 mismatch per index and only outputting reads that “pass filter”. Illumina’s pass filter algorithm screens out reads based on the signal intensities over the first 25 cycles of the sequencing read. The additional parameter “--create-fastq-for-index-reads” was specified to force the program to output fastq files for both index sequences (i5 and i7). Raw index and sequence reads are available from the sequence read archive (SRA) under accession number SRP083789. We also de-multiplexed another randomly selected index pair (i5: ACGTAAGG; i7: GGCCAATT) that was not used with any sample. This index pair had zero associated reads, confirming that the observed rates of sequence misassignment are larger than expected from misread bases alone.

Reads were assigned to the nearest expected sequence within an edit distance of four (including mismatches, insertions, and deletions) using the DECIPHER (v2.1.6) package [11] in R [12] (http://www.DECIPHER.codes). The sequences belonging to each sample were separated by an edit distance of at least 14, meaning that a small number of misread bases would not prevent correct matching. Barring insertions and deletions, which are uncommon on the Illumina platform, the 14 sequence variants were separated by between 21 and 43 substitutions. The probability of 17 (21 differences –4 mismatches) or more substitutions within 51 bases is 10^−23^ at a high misread rate of 1 % (Q20). Between 4.8 and 9.6 million reads were mapped to each of the 14 sequences having a known index pair, with a mean of 7.5 million reads per expected triplet. A total of 99.9 % of unexpected triplets differed from an expected triplet by a single read step, with the remainder differing by two read steps (e.g., incorrect i5 and i7).

Quality score filtering was applied with the TrimDNA function of DECIPHER [11], which allows specification of a maximum average error rate. The quality score (Q) can be converted to a probability of error (p) using the formula *p* = 10^(Q/−10)^. The sequence misassignment rate was calculated as the fraction of reads having the same i5 and i7 index pair that mapped to the wrong sequence, divided by the total number of mapped reads having that index pair. The index misassignment rate was calculated as the fraction of reads that mapped to a sequence with a known index pair, but differing by a single i5 or i7 index from the expected index pair.

## Additional file

Additional file 1: Figure S1. Average quality score per-base for each read type. **Tables S1 and S2**: Lists of strains and primers used in this study. (DOCX 450 kb)
