# Supplementary material for: Quality filtering of Illumina index reads mitigates sample cross-talk
Source: BMC Genomics. 2016 Nov 4;17:876. doi: 10.1186/s12864-016-3217-x (PMC5097354; doi:10.1186/s12864-016-3217-x)
Supplement: Additional file 1: Figure S1. — Average quality score per-base for each read type. Tables S1 and S2: Lists of strains and primers used in this study. (DOCX 450 kb) [file 12864_2016_3217_MOESM1_ESM.docx]

**Supplemental Figures**


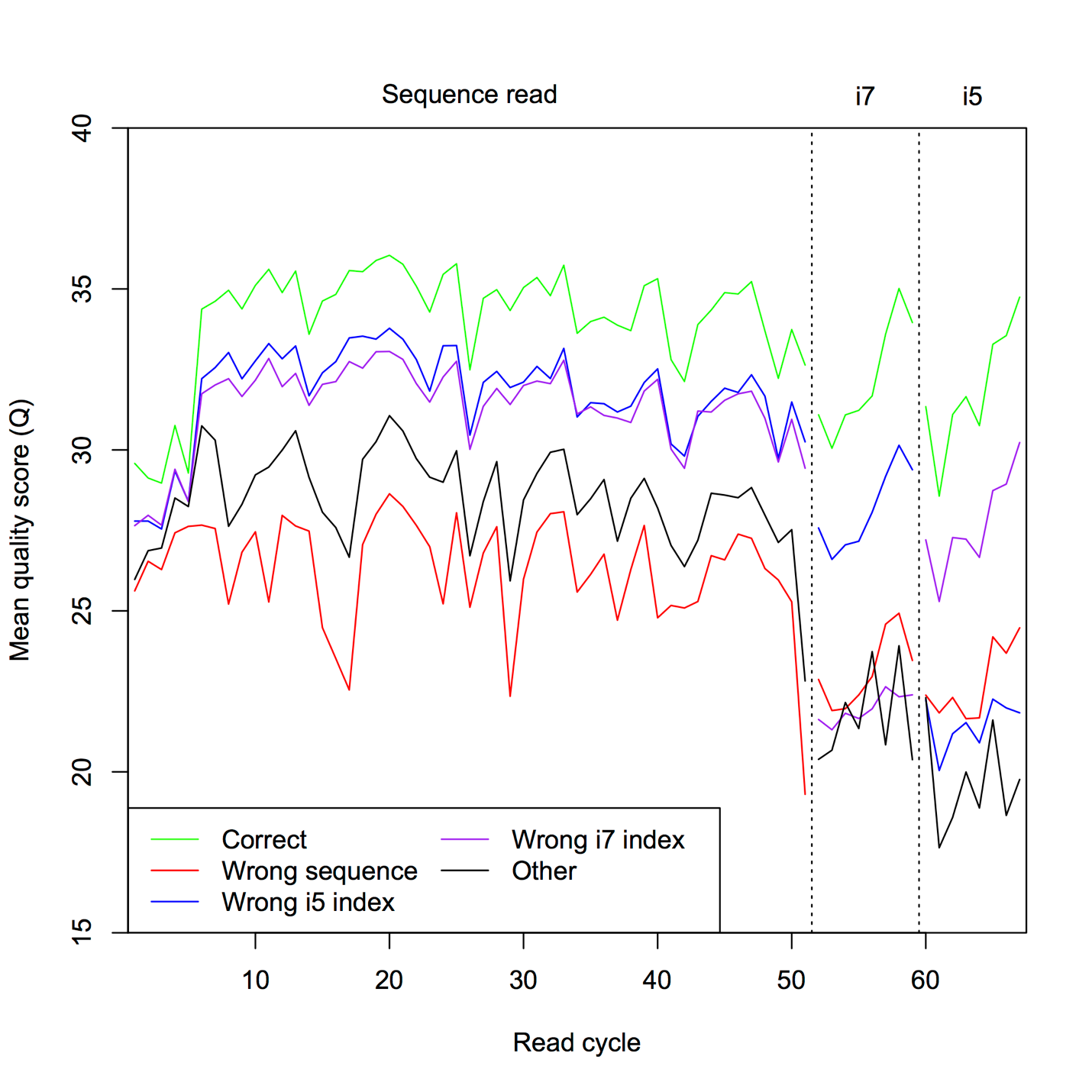


Supplemental Figure 1. Mean quality score per base for each read step (sequence, i7 index, or i5 index). The average quality was consistently lower across the entire length of the misassigned reads relative to correctly assigned reads. Furthermore, particular positions exhibited consistently lower scores across all read types, as well as across the 14 sequence variants.

**Supplemental Tables**

Supplemental Table 1

| **Target** | **Strain** | **Primer 1** | **Primer 2** | **i5 index** | **i7 index** |
| --- | --- | --- | --- | --- | --- |
| barcode | *Streptomyces lividans* | Left54 | Right28 | CAACGAAC | AGACGTTC |
| barcode | *Streptomyces S3H10* | Left55 | Right80 | CACACACT | ATGGTGTG |
| barcode | *Streptomyces coelicolor* | Left56 | Right66 | AATACCGC | ACCTACCA |
| barcode | *Streptomyces venezuelae* | Left41 | Right67 | AATGACGG | ACTTCGGT |
| barcode | *Streptomyces S26F9* | Left42 | Right29 | TGACGGAA | ACGGACTT |
| barcode | *Streptomyces albus J1074* | Left43 | Right81 | ACATGGCT | GCTGAACT |
| barcode | *Streptomyces G4A3* | Left78 | Right68 | CGGCTATT | CTACGCTA |
| barcode | *Streptomyces S25E2* | Left79 | Right30 | ATAGCGGT | CTAAGCGT |
| barcode | *Streptomyces S4B4* | Left57 | Right31 | CTGTTCGT | GATGTCCA |
| barcode | *Streptomyces S2D4* | Left44 | Right69 | CCGAATTG | AACACGAC |
| barcode | *Streptomyces S18A4* | Left45 | Right70 | TACTAGCG | GCGAGATT |
| *rpoB* | *Streptomyces S4B4* | Reverse3 | Forward1 | GAATTCGC | GCCTCTTA |
| *rpoB* | *Streptomyces cattelya* | Reverse25 | Forward2 | TTAGACCG | CAGCGTAT |
| *rpoB* | *Amycolatopsis AA4* | Reverse16 | Forward15 | TACCGAGT | TACCTCTG |

Supplemental Table 2

| **Primer Name** | **Primer Sequence (5' to 3')** |
| --- | --- |
| Forward1 | CAAGCAGAAGACGGCATACGAGATTAAGAGGCGTGACTGGAGTTCAGACGTGTGCTCTTCCGATCAAGGTCGGCCGCTACAAGGT |
| Forward15 | CAAGCAGAAGACGGCATACGAGATCAGAGGTAGTGACTGGAGTTCAGACGTGTGCTCTTCCGATCAAGGTCGGCCGCTACAAGGT |
| Forward2 | CAAGCAGAAGACGGCATACGAGATATACGCTGGTGACTGGAGTTCAGACGTGTGCTCTTCCGATCAAGGTCGGCCGCTACAAGGT |
| Left41 | AATGATACGGCGACCACCGAGATCTACACAATGACGGACACTCTTTCCCTACACGACG |
| Left42 | AATGATACGGCGACCACCGAGATCTACACTGACGGAAACACTCTTTCCCTACACGACG |
| Left43 | AATGATACGGCGACCACCGAGATCTACACACATGGCTACACTCTTTCCCTACACGACG |
| Left44 | AATGATACGGCGACCACCGAGATCTACACCCGAATTGACACTCTTTCCCTACACGACG |
| Left45 | AATGATACGGCGACCACCGAGATCTACACTACTAGCGACACTCTTTCCCTACACGACG |
| Left54 | AATGATACGGCGACCACCGAGATCTACACCAACGAACACACTCTTTCCCTACACGACG |
| Left55 | AATGATACGGCGACCACCGAGATCTACACCACACACTACACTCTTTCCCTACACGACG |
| Left56 | AATGATACGGCGACCACCGAGATCTACACAATACCGCACACTCTTTCCCTACACGACG |
| Left57 | AATGATACGGCGACCACCGAGATCTACACCTGTTCGTACACTCTTTCCCTACACGACG |
| Left78 | AATGATACGGCGACCACCGAGATCTACACCGGCTATTACACTCTTTCCCTACACGACG |
| Left79 | AATGATACGGCGACCACCGAGATCTACACATAGCGGTACACTCTTTCCCTACACGACG |
| Reverse16 | AATGATACGGCGACCACCGAGATCTACACTACCGAGTACACTCTTTCCCTACACGACGCTCTTCCGATCTACAGATGTCGTCGGTCTCGAC |
| Reverse25 | AATGATACGGCGACCACCGAGATCTACACTTAGACCGACACTCTTTCCCTACACGACGCTCTTCCGATCTGATGTCGTCGGTCTCGAC |
| Reverse3 | AATGATACGGCGACCACCGAGATCTACACGAATTCGCACACTCTTTCCCTACACGACGCTCTTCCGATCTCTGATGTCGTCGGTCTCGAC |
| Right28 | CAAGCAGAAGACGGCATACGAGATGAACGTCTGTGACTGGAGTTCAGACGTGTGCTCTTCCGATCTAGGCGATTAAGTTGGGTAACG |
| Right29 | CAAGCAGAAGACGGCATACGAGATAAGTCCGTGTGACTGGAGTTCAGACGTGTGCTCTTCCGATCTAGGCGATTAAGTTGGGTAACG |
| Right30 | CAAGCAGAAGACGGCATACGAGATACGCTTAGGTGACTGGAGTTCAGACGTGTGCTCTTCCGATCTAGGCGATTAAGTTGGGTAACG |
| Right31 | CAAGCAGAAGACGGCATACGAGATTGGACATCGTGACTGGAGTTCAGACGTGTGCTCTTCCGATCTAGGCGATTAAGTTGGGTAACG |
| Right66 | CAAGCAGAAGACGGCATACGAGATTGGTAGGTGTGACTGGAGTTCAGACGTGTGCTCTTCCGATCTAGGCGATTAAGTTGGGTAACG |
| Right67 | CAAGCAGAAGACGGCATACGAGATACCGAAGTGTGACTGGAGTTCAGACGTGTGCTCTTCCGATCTAGGCGATTAAGTTGGGTAACG |
| Right68 | CAAGCAGAAGACGGCATACGAGATTAGCGTAGGTGACTGGAGTTCAGACGTGTGCTCTTCCGATCTAGGCGATTAAGTTGGGTAACG |
| Right69 | CAAGCAGAAGACGGCATACGAGATGTCGTGTTGTGACTGGAGTTCAGACGTGTGCTCTTCCGATCTAGGCGATTAAGTTGGGTAACG |
| Right70 | CAAGCAGAAGACGGCATACGAGATAATCTCGCGTGACTGGAGTTCAGACGTGTGCTCTTCCGATCTAGGCGATTAAGTTGGGTAACG |
| Right80 | CAAGCAGAAGACGGCATACGAGATCACACCATGTGACTGGAGTTCAGACGTGTGCTCTTCCGATCTAGGCGATTAAGTTGGGTAACG |
| Right81 | CAAGCAGAAGACGGCATACGAGATAGTTCAGCGTGACTGGAGTTCAGACGTGTGCTCTTCCGATCTAGGCGATTAAGTTGGGTAACG |
